# Theranostic Studies of Human Sodium Iodide Symporter Imaging and Therapy Using ^188^Re: A Human Glioma Study in Mice

**DOI:** 10.1371/journal.pone.0102011

**Published:** 2014-07-07

**Authors:** Rui Guo, M. Zhang, Yun Xi, Yufei Ma, Sheng Liang, Shuo Shi, Ying Miao, Biao Li

**Affiliations:** 1 Department of Nuclear Medicine, Rui Jin Hospital, School of medicine, Shanghai JiaoTong University, Shanghai, China; 2 Department of Nuclear Medicine, Xin Hua Hospital, School of medicine, Shanghai JiaoTong University, Shanghai, China; Technische Universitaet Muenchen, Germany

## Abstract

**Objective:**

To investigate the role of ^188^Re in human sodium iodide symporter (hNIS) theranostic gene-mediated human glioma imaging and therapy in model mice.

**Methods:**

The human glioma cell line U87 was transfected with recombinant lentivirus encoding the hNIS gene under the control of cytomegalovirus promoter (U87-hNIS). The uptake and efflux of ^188^Re were determined after incubating the cells with ^188^Re. ^188^Re uptake experiments in the presence of various concentrations of sodium perchlorate were carried out. In vitro cell killing tests with ^188^Re were performed. U87-hNIS mediated ^188^Re distribution, imaging and therapy in nude mice were also tested.

**Results:**

U87-hNIS cell line was successfully established. The uptake of ^188^Re in U87-hNIS cells increased up to 26-fold compared to control cells, but was released rapidly with a half-life of approximately 4 minutes. Sodium perchlorate reduced hNIS-mediated ^188^Re uptake to levels of control cell lines. U87-hNIS cells were selectively killed following exposure to ^188^Re, with a survival of 21.4%, while control cells had a survival of 92.1%. Unlike in vitro studies, U87-hNIS tumor showed a markedly increased ^188^Re retention even 48 hours after ^188^Re injection. In the therapy study, there was a significant difference in tumor size between U87-hNIS mice (317±67 mm^3^) and control mice (861±153 mm^3^) treated with ^188^Re for 4 weeks (*P*<0.01).

**Conclusion:**

The results indicate that inserting the hNIS gene into U87 cells is sufficient to induce specific ^188^Re uptake, which has a cell killing effect both in vitro and in vivo. Moreover, our study, based on the function of hNIS as a theranostic gene allowing noninvasive imaging of hNIS expression by ^188^Re scintigraphy, provides detailed characterization of in vivo vector biodistribution and level, localization, essential prerequisites for precise planning and monitoring of clinical gene therapy that aims to individualize gene therapy concept.

## Introduction

Glioma remains one of the most common cancers and is a leading cause of cancer-related deaths worldwide. Furthermore, glioma carries a poor prognosis and survival rate. Aside from tumor resection, radiotherapy is the major curative therapy for glioma. However, patients often develop normal brain tissue necrosis following external radiation [1]; thus, new therapeutic strategies are required. The theranostic strategy [2], [3] using radionuclide-based imaging reporter genes shows great treatment promise for various clinical fields, particularly in the field of cancer gene therapy.

As a theranostic gene, the sodium iodide symporter (NIS) is a plasma membrane glycoprotein, which mediates active iodide uptake in the thyroid and other tissues [4], [5]. One of the most exciting current areas of NIS research is radioiodine treatment of extrathyroidal cancers by the ectopic transfer of the NIS gene into otherwise non-NIS-expressing cancers. Many investigators have successfully obtained ectopic NIS expression by gene transfer techniques in prostate cancer [6], melanoma [7], glioma cells [8] and myeloma cells [9].

Our previous studies [10]–[13] suggest that baculovirus mediate human NIS (hNIS) expression leads to ^131^I uptake in several types of tumors and presents as a promising target for gene therapy. Although the baculovirus can mediate gene transduction effectively and achieve desirable expression in various tumor cell lines in vitro, there are still some obstacles to overcome concerning the in vivo application of this system in gene therapy. For example, a major concern is the inactivation of baculovirus by the serum complement in baculovirus-based gene therapy in vivo.

In previous studies [14]–[15], extrathyroidal tissues are generally not able to organify iodide after NIS gene transfer. In the contrast, ^131^I accumulates and is organified in the thyroid, which exhibits competitive inhibition in extrathyroidal tumor ^131^I uptake, preventing the delivery of a radiation dose high enough to affect cell viability; therefore, the therapeutic efficacy of ^131^I is limited [16]. The application of alternative radioisotopes that are also transported by hNIS with a shorter physical half-life and a high energy to ^131^I may provide a powerful method for enhancing the therapeutic efficacy of hNIS-targeted radionuclide therapy [17], [18]. ^188^Re is a β-emitting radionuclide with a short physical half-life (0.71 day) that has been used in a variety of therapeutic applications in humans, including cancer radioimmunotherapy and palliation of skeletal bone pain [19]. Due to its higher relative energy compared to ^131^I, administration of ^188^Re offers the possibility of higher energy deposition over a shorter time period. Compared to ^131^I (E = 0.192 MeV),^ 188^Re has been proposed as an ideal alternative emitter (E = 0.778 MeV) to ^131^I for cancer treatment. Kang et al [20] investigated ^188^Re accumulation of a human hepatocellular carcinoma cell line, SK-Hep1, by transfer of human sodium iodide symporter (hNIS) gene and found it has the potential to be used in hepatocellular carcinoma management. To date, no studies have explored whether lentivirus-mediated hNIS gene expression and ^188^Re uptake can be used for glioma imaging and therapy. In this study, we investigated the role of ^188^Re as a potential alternative radionuclide for hNIS-mediated imaging and treatment of human glioma in model mice.

## Materials and Methods

### Plasmid construction, lentivirus preparation, and U87 cell transfection with Lenti-CMV-hNIS

The human NIS gene was removed from the pcDNA3.1-hNIS vector (kindly provided by Dr. Sissy Jhiang from Ohio University, OH, USA) by restriction enzyme digestion. All plasmid construction and lentivirus (Lenti-CMV-hNIS) production was performed according to Life Technologies (Carlsbad, CA, USA) manufacturing instructions. Lenti-CMV-0 with no hNIS was prepared as a control. U87 human glioma cell line (American Type Culture Collection, AT CC) were maintained in Dulbecco’s modified Eagle’s medium (DMEM) supplemented with 10% fetal bovine serum, 5% CO_2_, 37°C, 100 U/ml penicillin and 100 µg/ml streptomycin. To generate cell lines expressing hNIS controlled by the cytomegalovirus (CMV)-enhancer/promoter, the Lenti-CMV-hNIS vector was transfected into U87 cells and selected for weeks according to the manufacturer’s instructions. Established cells (U87-hNIS) were confirmed through ^188^Re uptake experiments. U87-0 cells transfected with Lenti-CMV-0 (hNIS gene negative) were prepared as controls. Cells were maintained in Dulbecco’s modified eagle medium (DMEM; Life Technologies) supplemented with fetal bovine serum (10%), and maintained at 37°C in a humidified atmosphere with 5% CO_2_. Cells were seeded in 24-well plates 24 hours before experiments to achieve a density of 10^5^ cells/well at the day of study.

### *In vitro* isotope uptake experiments

^188^Re was eluted from a ^188^W/^188^Re generator (LaiTai Company, Suzhou, People’s Republic of China) using 0.9% saline. hNIS expression in U87-hNIS cells was tested in vitro by ^188^Re uptake as Weiss et al. described previously with minor modifications [21]. All activity data were corrected for decay and normalized. U87-hNIS cells were washed with 0.5 mL Hank’s balanced salt solution (bHBSS; HBSS supplemented with 10 µM sodium iodide and buffered with Hepes, pH 7.3) once, and incubated for 1, 2, 5, 10, 20, 30, 60, and 120 minutes, respectively, in the presence of 0.5 mL bHBSS containing 3.7 kBq^ 188^Re. The cells were then washed twice with ice-cold bHBSS and incubated with 1 mL of 100% ice-cold dehydrated alcohol for 20 minutes. The radioactivity (counts per minute [cpm]) in cell lysates was measured using a multi-well gamma-counter (Shanghai Institute of Nuclear Research Rihuan Instrument Company, China). All experiments were performed in triplicate. U87-0 transfected cells were prepared as a control.

### Sodium perchlorate inhibition study

We used a sodium perchlorate inhibition study to test the specificity of ^188^Re uptake. U87-hNIS cells were incubated for 30 minutes in 3.7 kBq ^188^Re medium supplemented with sodium perchlorate (Sigma-Aldrich, St. Louis, MI, USA) at concentrations of 0, 1 µM, 2 µM, 5 µM, 10 µM, 20 µM, 50 µM, and 100 µM. Then the cells were washed, lysed, and counted as described previously.

### Isotope efflux study *in vitro*

We evaluated ^188^Re efflux kinetics in cells expressing hNIS as previously described by Weiss et al [21]. Briefly, U87-hNIS cells were exposed to 3.7 kBq/well ^188^Re at 37°C for 20 minutes. Cell medium was then replaced with fresh, nonradioactive bHBSS. The cells were incubated for 2, 4, 6, 8, 10, 12, 14, 16, 18 or 20 min and immediately lysed as described previously. After the incubation period, the cells were extracted with 1 mL of dehydrated alcohol, and the residual activity in the cells were measured.

### *In vitro* assessment of isotope toxicity by clonogenic assay

U87-hNIS and U87-0 cells were seeded in 24-well plates 24 hours before the experiment to achieve a density of 10^5^ cells/well on the day of study. As shown in Table 1, cells were divided into six groups: Group 1 included U87-hNIS cells washed with bHBSS and allowed to incubate for 7 hours (5% CO_2_, 37°C) after the addition of 740 kBq ^188^Re/mL; Group 2 was processed as Group 1, but without addition of ^188^Re; Group 3 included U87-0 cells washed with bHBSS and allowed to incubate for 7 hours after the addition of 740 kBq ^188^Re/mL; Group 4 was processed as Group 3, but without addition of ^188^Re; Group 5 included non-transfected U87 cells that were washed with bHBSS and allowed to incubate for 7 hours after the addition of 740 kBq ^188^Re/mL; Group 6 was processed as Group 5, but without addition of ^188^Re. Groups 2, 3, 4, 5 and 6 all served as controls. The six groups of cells were then washed twice with bHBSS, trypsinized, counted, and plated at a density of 200 cells/well in 6-well plates in triplicate. The cells were placed in 5% CO_2_ at 37°C for 7 days. After removing the culture medium, each plate was stained with crystal violet solution (0.1%), and colonies including more than 30 cells were counted. Results are expressed as the percentage of surviving cells, ie, the percentage of colonies obtained after treatment with lentivirus and/or ^188^Re compared to treatment with bHBSS alone (Group 6).

**Table 1:** The subgroups used in the in^188^Re clonogenic assay.

	Group1	Group2	Group3	Group4	Group5	Group6
U87-hNIS	+	+				
U87-0			+	+		
U87					+	+
^188^Re	+		+		+	

### Biodistribution of ^188^Re in xenografted mice

For biodistribution studies, the U87-hNIS tumor xenografted mice were randomly divided into 4 groups with 6 mice per group. Each animal received a 370 kBq intravenous (i.v.) injection of ^188^Re to evaluate biodistribution of the tracer in the tumor and major organs. The mice were sacrificed 0.5 h, 2 h, 12 h and 24 h after the radionuclide injection, the main organs and xenografted tumors were removed, weighed, and counted for radioactivity by using a gamma counter. All tissue counts were corrected for background and decay during the time of counting. Results were expressed as a percentage of the injected dose per gram (% ID/g) of tissue. Each value represents the mean and SD of 6 animals. The U87-0 tumor xenografted mice were served as controls and processed as described above.

### Tumor imaging and therapy study in a xenograft model

Animal procedures were carried out following the approval of the Ethics Committee and Animal Care Committee of Shanghai Jiaotong University, School of Medicine. Five-week-old female, athymic Balb/c nude mice were used in the following experiments; six mice were used in each group. A xenograft model was generated by subcutaneous injection of U87-hNIS or U87-0 cells (5×10^6^ cells suspended in 150 µL phosphate buffered saline) into the right axilla of the mice. Approximately 6 weeks after inoculation when the tumor diameter reached 0.8–1.0 cm, the mice were used for imaging and therapeutic studies.

Xenograft models of U87-hNIS and U87-0 cells were established (n = 6 for each group). ^188^Re (55.5 MBq) was injected through the caudal vein. Immediately after intravenous injection of ^188^Re into the mice, the time dependent accumulation of radioactivity in the mice was monitored 15 minutes, 0.5, 1, 2, 4, 24, and 48 hours after injection using a γ-camera (GE Healthcare, Cleveland, OH, USA), equipped with a high-resolution pinhole collimator with a matrix size of 256×256. Elliptical regions of interest (ROI) were placed on the tumor and on the contralateral armpit region, from which tumor-to-nontumor (T/NT) contralateral armpit count ratios of each group were measured and calculated in every time point.

To evaluate the in vivo effects of hNIS-mediated radioiodine therapy, U87-hNIS tumor size of xenograft models were measured before and after the intravenous injection of 18.5 MBq, 55.5 MBq and 111 MBq^ 188^Re respectively (n = 6 for each group). U87-0 tumor xenograft models were treated with 55.5 MBq ^188^Re and served as controls. All mice were followed for a total of 4 weeks. Tumor volume was estimated using the following formula: length×width^2^×0.52.

### Statistical analysis

Origin 7.5 (OriginLab Corporation, Northampton, MA, USA) and SPSS 16.0 (IBM Corporation, Armonk, NY, USA) were used for all statistical analyses. Numeric data are expressed as means ± standard deviation (SD). Data comparisons between groups were performed by analysis of variance (ANOVA) test. *P* values*<*0.05 were considered statistically significant.

## Results

### Plasmid construction, lentivirus preparation, and U87 cell transfection with Lenti-CMV-hNIS

We successfully developed a lentivirus-derived vector containing the hNIS gene under the control of the CMV promoter and yielded virus (Lenti-CMV-hNIS) stocks. Lenti-CMV-0 was also prepared as control with the same titer. Approximately 3 weeks later, after infection of U87 cells with the recombinant lentiviruses, stable cell lines U87-hNIS and U87-0 were established. In order to investigate the function of hNIS in the recombinant U87-hNIS cell lines, ^188^Re uptake experiments were performed.

### hNIS-mediated *in vitro* ^188^Re uptake

**Figure 1:**
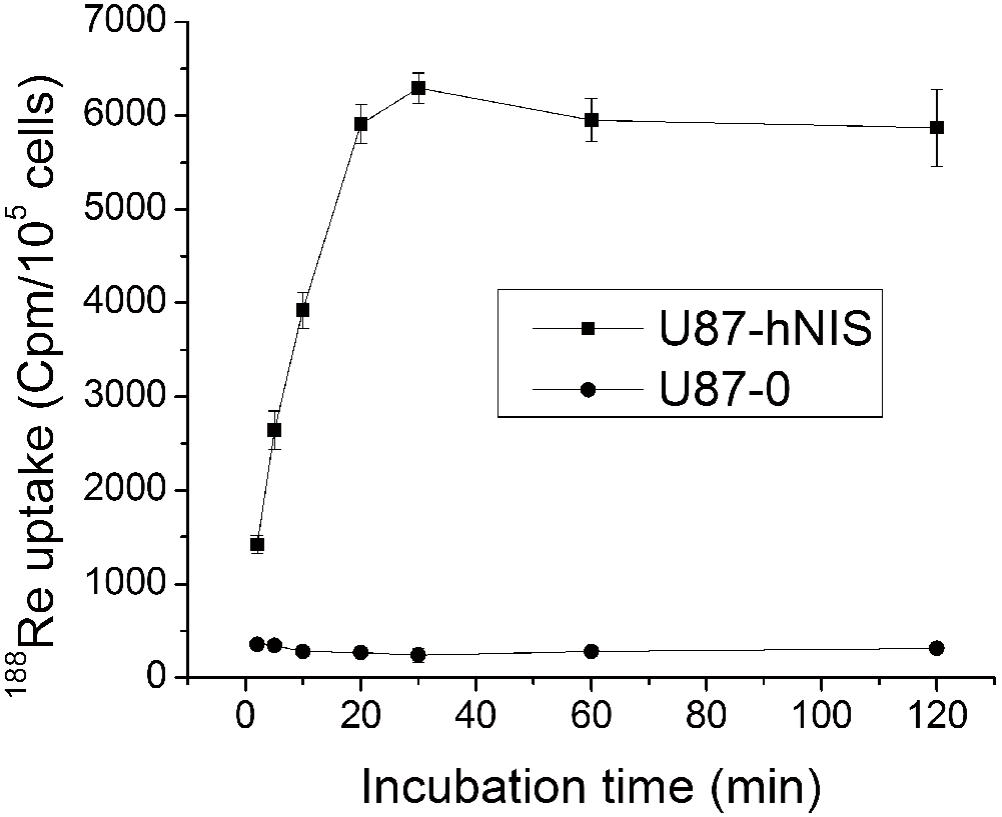
Time dependent ^188^Re uptake by U87-hNIS cells. ^188^Re uptake was measured by incubation for various time periods with 3.7 kBq of ^188^Re in bHBSS. Results are expressed in numbers of counts per minute for 10^5^ cells. The upper line represents U87-hNIS cells, while the lower line represents U87-0 cells. Data points are means ± SD (n = 3).

^188^Re uptake in U87-hNIS cells with respect to ^188^Re incubation time is shown in Figure 1; the initial uptake of ^188^Re was dependent on incubation time. ^188^Re influx rapidly increased into U87-hNIS cells, with half-maximal uptake observed at about 5 minutes, reaching a maximum after about 30 minutes. ^188^Re uptake in U87-hNIS cells was 26-fold higher than in U87-0 cells after incubation with ^188^Re for 30 minutes. At that time point, ^188^Re accumulation achieved a peak; therefore, 30 minutes represented the U87-hNIS maximal ^188^Re uptake and was selected as the incubation time for subsequent experiments. The intracellular radioactivity was calculated to be up to 3.3% of the total radioactivity in cell lysate and media. Assuming that U87-hNIS cells have a mean diameter of 10 µm, we estimated that an up to 328-fold higher concentration of ^188^Re was observed in cells compared to media. The radioactivity measured 2 hours after ^188^Re incubation was 93.3% of the maximal uptake. There is a plateau, as Zuckier et al. [22] reported in similar studies, which suggest steady-state uptake.

### Sodium perchlorate inhibition study

**Figure 2:**
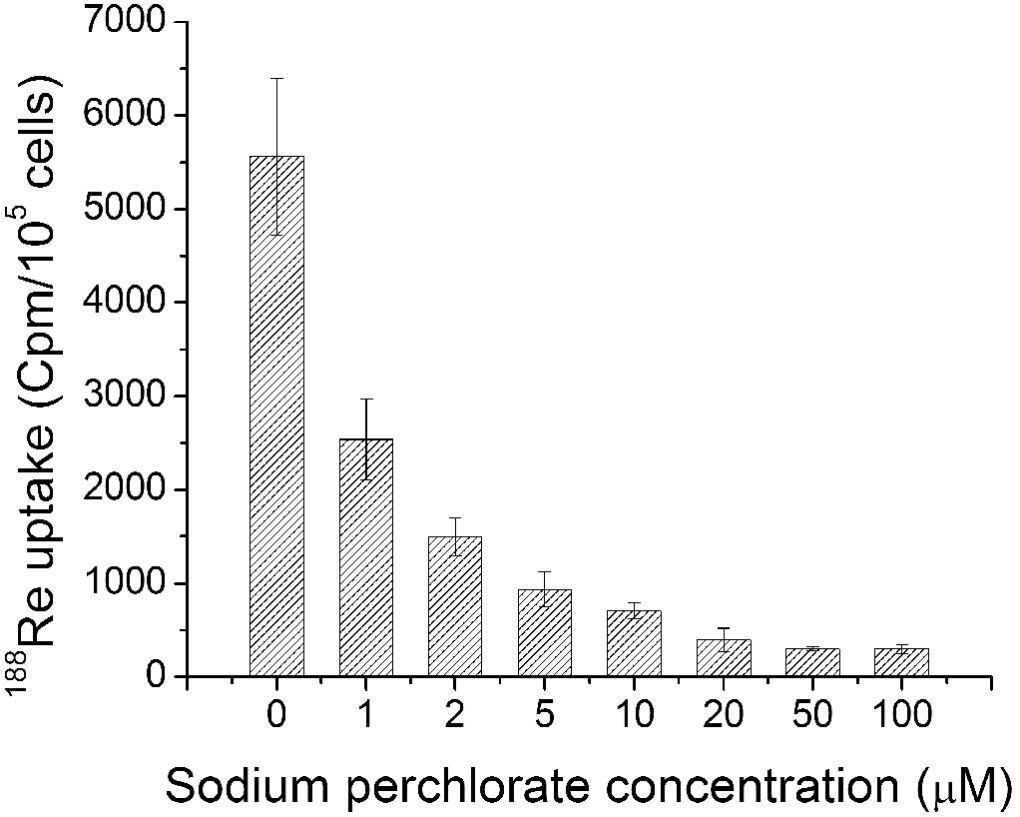
Sodium perchlorate inhibition study. Sodium perchlorate was added at the indicated concentration (0, 1, 2, 5, 10, 20, 50, and 100 µM) and^ 188^Re uptake was measured. Results are expressed in numbers of counts per minute for 10^5^ cells. Bars are means ± SD (n = 3).

To evaluate whether ^188^Re accumulation was specifically induced by the functional activity of the hNIS gene product, ^188^Re uptake was determined in U87-hNIS cells in the presence of various concentrations of sodium perchlorate, an established competitive inhibitor. Figure 2 shows the effect of sodium perchlorate on ^188^Re uptake in U87-hNIS cells. Uptake of ^188^Re showed dose-dependent inhibition in U87-hNIS cells in experiments performed within a range of 1–50 µM NaClO_4_; 5 µM NaClO_4_ decreased ^188^Re uptake to 16.8%, while at a concentration of 50 µM, sodium perchlorate inhibited ^188^Re accumulation in U87-hNIS cells by 94.6%. ^188^Re uptake was blocked and significantly decreased in the presence of sodium perchlorate, indicating that ^188^Re is uptaken through functional hNIS in U87-hNIS cells.

### Radionuclide efflux assay

**Figure 3:**
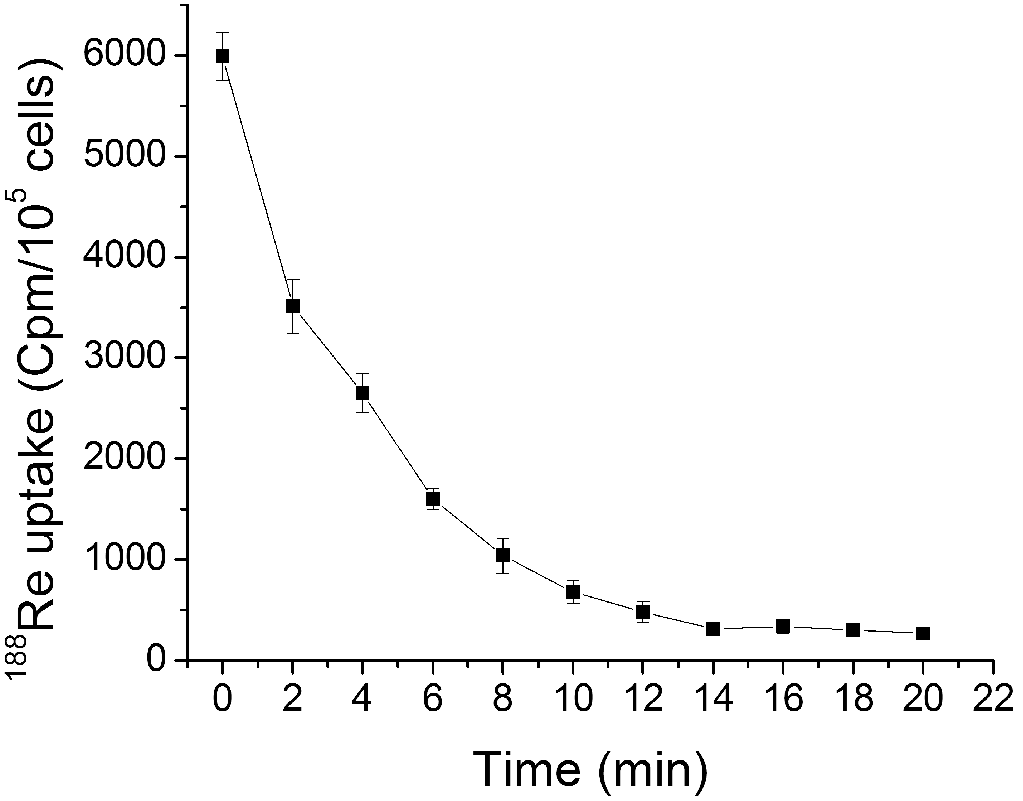
Time dependent^ 188^Re efflux assay in U87-hNIS cells after a 20 minute incubation with 3.7 kBq ^188^Re in 0.5 mL bHBSS. Results are expressed as counts per minute for 105 cells. Data points are means ± SD (n = 3).

On reaching maximum ^188^Re uptake after approximately 30 min, the ^188^Re efflux assay was continued for 20 min (Figure 1). As shown in Figure 3, the amount of remaining ^188^Re in U87-hNIS cell lysate was determined as a function of time after replacement of ^188^Re containing media with nonradioactive media. The cellular radioactivity was rapidly and continuously released into the media, with 55.7% of the total cellular ^188^Re released within 4 minutes, and 95.5% efflux was observed after 20 minutes, indicating that the radiotracer was rarely trapped in U87-hNIS cells. Accordingly, under in vitro conditions limiting re-uptake, a rapid release of ^188^Re was observed.

### ^188^Re toxicity assessed by colony-forming assay

**Figure 4:**
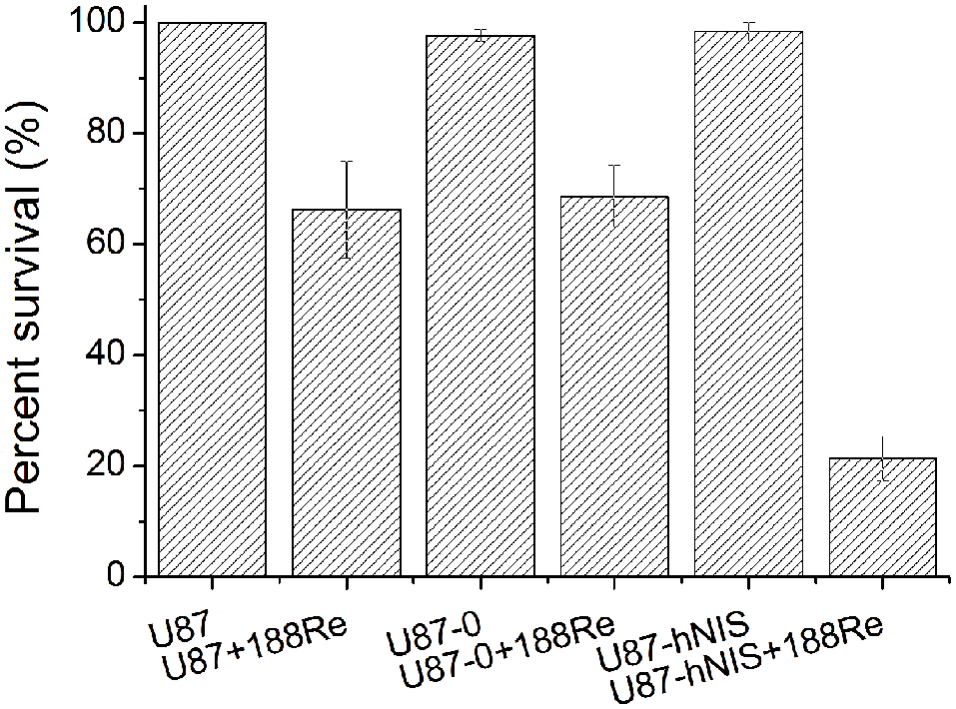
Survival rates of U87-hNIS cells treated with 740 kBq ^188^Re/mL ^188^Re. Groups 2, 3, 4, 5, and 6 all served as controls. Bars are means ± SD (n = 3).

In vitro^ 188^Re toxicity experiments were performed on U87-hNIS cells to demonstrate whether it was possible to obtain a cell killing effect with ^188^Re. After ^188^Re treatment, clonogenic assays were performed, the results of which are shown in Figure 4. The data is expressed as the percentage of surviving cells. In Group 2 and Group 4, U87-hNIS and U87-0 cells treated without ^188^Re, the numbers of colonies were 98.3% and 97.7% of blank cells (Group 6), respectively, indicating that transfection with the lentivirus did not affect cell survival. In Group 3 and Group 5, after exposure to ^188^Re, 68.7% and 66.3% of the cells survived, respectively. In Group 1, the number of ^188^Re treated U87-hNIS colonies that recovered were significantly lower than in the other five groups, with a survival of about 21.3%, demonstrating a selective killing effect of ^188^Re in hNIS-expressing cells, which is the end goal of this system.

### Biodistribution of ^188^Re in xenografted mice

**Figure 5:**
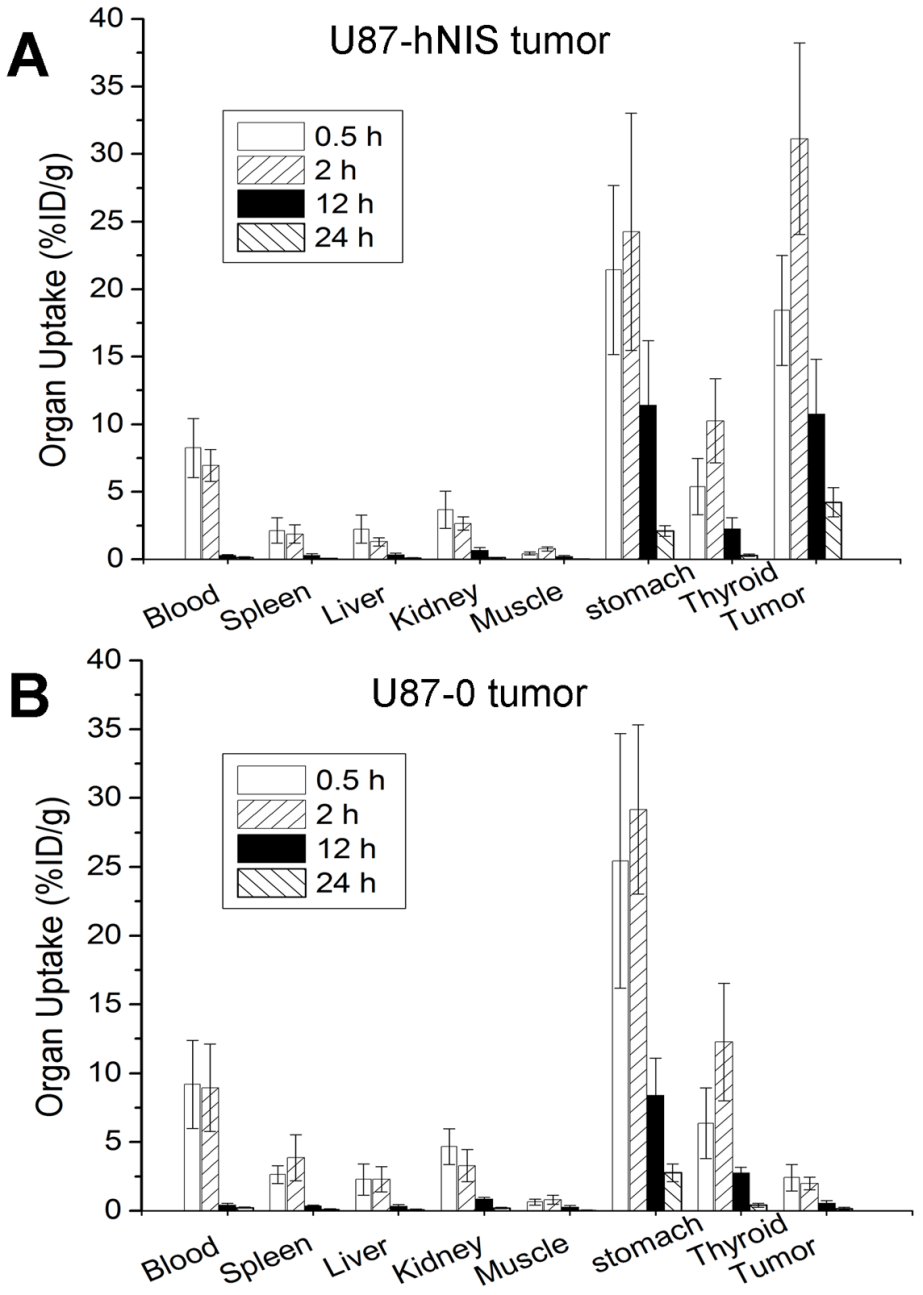
Biodistribution of ^188^Re in nude mice bearing subcutaneously xenografted U87-hNIS glioma tumors (A) and U87-0 glioma tumors (B). Data are expressed as the % ID/g of tissue. Bars are the mean ± standard deviation (n = 6).

The biodistribution data of ^188^Re in U87-hNIS tumor xenografted models are illustrated in Fig. 5A. The radiotracer exhibited a rapid decrease in radioactivity over time in blood and most organs. The high activity of stomach was evidently attributable to the excretion of ^188^Re in the gastric mucosa. The highest tumor uptake of ^188^Re (31.13±7.09% ID/g) was found 2 h after injection and keeped to 10.72±4.09% ID/g 12 h after injection, even 4.23±1.07% ID/g 24 h after injection, suggesting that the ^188^Re was present in the U87-hNIS tumor up to at least 24 h after injection. While in U87-0 tumor xenografted mice, the tumor showed little ^188^Re uptake. The highest tumor uptake of ^188^Re (2.41±0.97% ID/g) was found 0.5 h after injection and decreased to 0.57±0.19% ID/g 12 h after injection. There is rarely ^188^Re retention (0.17±0.08% ID/g) 24 h after injection, suggesting that little ^188^Re was present in the U87-0 tumor.

### Tumor imaging and therapy study

**Figure 6:**
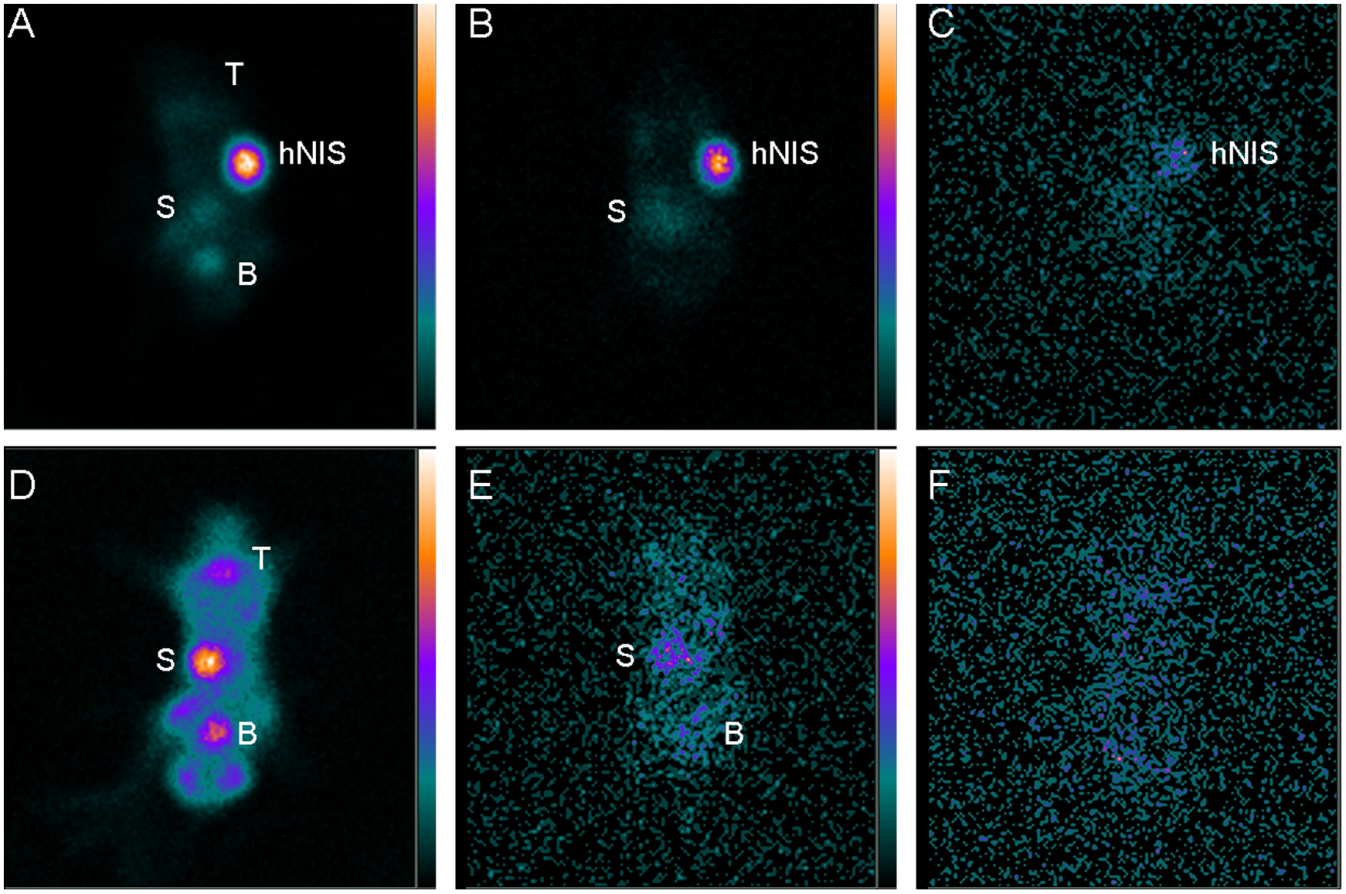
Whole-body scintigraphic images (anterior view) of hNIS-expressing U87-hNIS tumors (the first line, right axilla) or U87-0 tumors (the second line, right axilla) at 1-hour (A, D), 24 hours (B, E), and 48 hours (C, F) after intravenous injection of ^188^Re. T = thyroid; S = stomach; B = bladder; hNIS = hNIS-expressing tumor.

**Figure 7:**
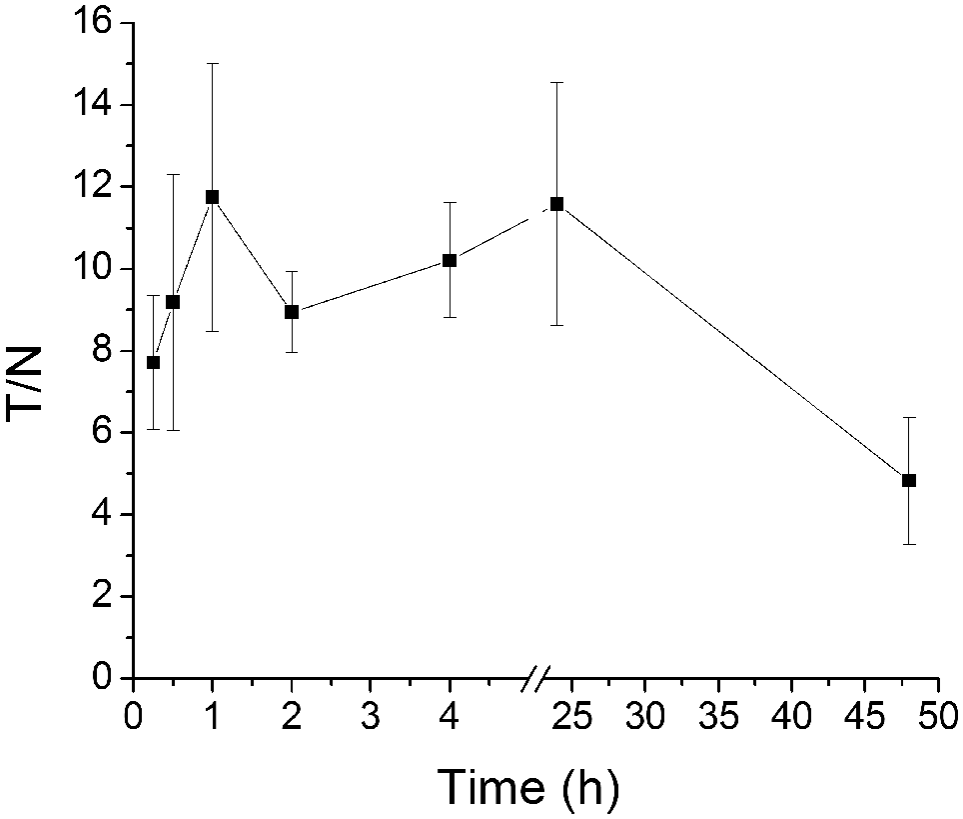
Time dependent tumor/non-tumor (T/NT) ratio of ^188^Re uptake by U87-hNIS tumor bearing nude mice. Data points are means ± SD (n = 6).

^188^Re uptake and efflux in vivo was not consistent with the data obtained from the in vitro studies. For instance, U87-hNIS tumors showed efficient ^188^Re uptake in vivo, and ^188^Re rapidly and significantly accumulated in the right axilla of mice (Figure 6A, B, C), leading to scintigraphic visualization, whereas the U87-0 control tumors were not visible (Figure 6D, E, F). As expected, tissues naturally expressed NIS (in the thyroid and stomach, for example) or in organs involved in ^188^Re elimination (such as the bladder) were also visualized, but significantly lower relative to tumor uptake. In U87-hNIS tumors, as shown in Figure 7, the tracer accumulation increased to maximum levels 1-hour after administration (T/NT 11.75±3.27), remained steady up to 24 hours (T/NT 11.58±2.96), and ^188^Re remained in U87-hNIS tumors even 48 hours later (T/NT 4.83±1.55). These are consistent with the results of biodistribution studies in U87-hNIS tumor xenografts. Furthermore, little ^188^Re is retained in the thyroid gland; the reason for this is that ^188^Re cannot be organified by the thyroid gland, which alongside with its shorter half-life, should substantially reduce the risk of post-treatment hypothyroidism.

**Figure 8:**
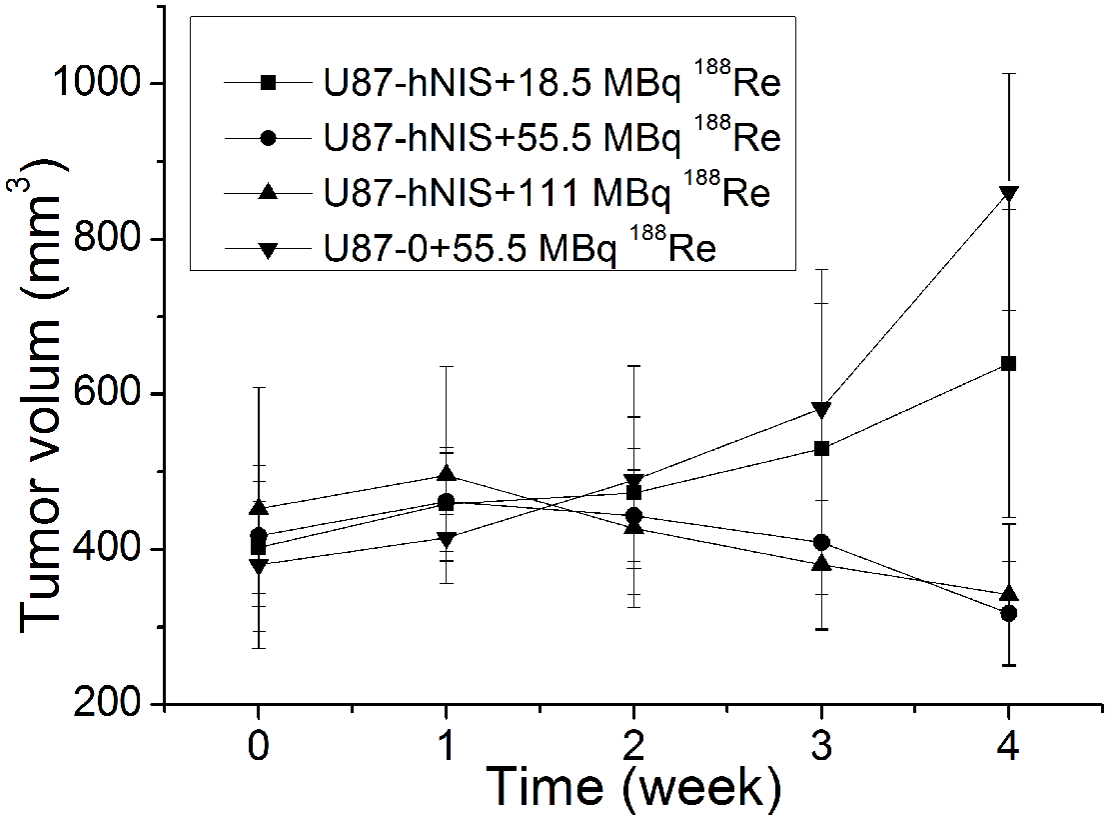
Human NIS induced ^188^Re therapy in U87-hNIS tumor bearing nude mice; 18.5 MBq, 55.5 MBq and 111 MBq of ^188^Re were injected intravenously in each mouse. The tumor size was measured before and after ^188^Re administration. U87-0 tumor bearing nude mice were severd as controls. Data points are means ± SD (n = 6).

As showen in Figure 8, although U87-hNIS tumors treated with 18.5 MBq ^188^Re showed no significant shrinkage, the tumor growth was retarded significantly 4 weeks after treatment, whereas the U87-0 tumors treated with 55.5 MBq ^188^Re rapidly increased in size. A significant difference in tumor size could be observed in U87-hNIS tumors treated with 18.5 MBq ^188^Re (640±199 mm^3^) compared to U87-0 tumors treated with 55.5 MBq ^188^Re (861±153 mm^3^) after 4 weeks of ^188^Re treatment (*P<*0.05). The difference of tumor volume between U87-hNIS tumors treated with 18.5 MBq ^188^Re (640±199 mm^3^) and 55.5 MBq ^188^Re (317±67 mm^3^) was statistically significant (*P<*0.01). While the difference of tumor volume between U87-hNIS tumors treated with 111 MBq ^188^Re (342±90 mm^3^) and 55.5 MBq ^188^Re (317±67 mm^3^) showed no statistical significance (*P>0.05*).

## Discussion

Most gliomas are resistant to currently available chemotherapy regimens. Besides tumor resection, external radiotherapy is a major curative therapy for glioma. However, patients are often either not responsive to or suffer from side effects from these conventional therapies. Radionuclide-based theranostic strategies have been widely used in the diagnosis and treatment of patients with hyperthyroidism or differentiated thyroid cancer, and the sodium iodide symporter gene is the radionuclide-based reporter gene used in theranostics. Theranostics is a promising approach offering the ideal combination of accurate diagnosis and successful therapy in various clinical fields, which is expected to become a key area of personalized medicine in the near future. In order to attain the ultimate goals of personalized medicine, which is to provide the highest therapeutic effect and to avoid adverse effects for each patient, a tailored therapeutic plan should be developed by obtaining accurate, detailed diagnostic information regarding the patient’s unique circumstances. Theranostics are an example of rapid advancement in biotechnologies for use with theranostic reporter genes and theranostic radiochemistry, which has led to the development of the concept of using theranostics with radionuclide-based imaging reporter genes [3].

NIS-mediated radionuclide therapy has several features that make it an attractive theranostic approach for the imaging and treatment of gliomas. For instance, complex radiolabeling procedures are not required for NIS-mediated radionuclide therapy. The small sizes of NIS radioactive substrates should result in both increased penetration of the blood-brain barrier and better diffusion capacity within the tumor. Radioisotopes have the potential of a bystander effect, in that tumor cells that do not express NIS can still be destroyed by electrons emitted from the surrounding, transduced tumor cells that express NIS and concentrate the isotope [23].

The traditionally used ^131^I after NIS gene transfer demonstrates limited therapeutic efficacy due to rapid iodide efflux. A strategy to enhance the therapeutic efficacy of NIS-targeted radionuclide therapies in tumors with rapid iodide efflux might be the application of more potent isotopes, such as ^188^Re, which are also transported by NIS, but in contrast to ^131^I offer the possibility of higher energy deposition in the tumor over a shorter period of time due to its shorter physical half-life and higher energy; together, this suggests a superior therapeutic efficacy in medium or large tumors by an enhanced “crossfire effect”. In this study, we explored an alternative method of hNIS-mediated therapy using ^188^Re. As can be seen from our in vivo study results, the degree of uptake and retention is sufficient for delivery of therapeutic doses of radiation to NIS-expressing tumors, considering the average energy of ^188^Re (E = 0.778 MeV vs. 0.192 MeV for ^131^I, which is 4.05-fold higher than that of ^131^I) and its considerably shorter physical half-life (0.71 days vs. 8.02 days for ^131^I) [23]. These properties make ^188^Re a worthy candidate for investigating its therapeutic efficacy after targeted NIS gene transfer in nonthyroidal cancers.

In our study, we demonstrated that ^188^Re uptake was very rapid in U87-hNIS cells; the initial kinetic of ^188^Re uptake was similar to what is observed in other virus transfected cells [24], [25], reaching a maximum concentration after about 30 minutes. ^188^Re accumulation was 26-fold higher compared to U87-0 control cells. There is a plateau phase, as is demonstrated in other studies [24], [25], which represents the steady-state of transport processes when influx and efflux are balanced [21]. Similar to other NIS related studies, relatively low radionuclide retention was a problem in our in vitro study; the amount of ^188^Re retained in U87-hNIS cells decreased significantly. However, U87-hNIS cells were efficiently killed by ^188^Re, as revealed by clonogenic assays. In our study, the absorbed dose of ^188^Re was sufficient for a significant selective killing effect of 78.7% using ^188^Re in an in vitro clonogenic assay, while U87-0 cells showed a non-selective killing effect of approximately 31.3%.

In this context, it is also important to mention that the in vitro monolayer system is an artificial system and does not allow the full assessment of the therapeutic efficacy of a radionuclide due to the lack of a three-dimensional structure, which requires further exploration in in vivo xenotransplant models. Dinglia et al. [26] suggested that therapy depends on adequate retention of the isotope in the tumor. In the absence of iodide organification, isotope trapping is a dynamic process either due to slow efflux or re-uptake of the isotope by cells expressing NIS. With sufficient NIS expression, iodide efflux is a zero-order process and iodide organification is insignificant. In our in vivo imaging study, ^188^Re remained in the U87-hNIS tumor even 48 hours after administration. In the following therapy study, there was a significant difference in tumor size between U87-hNIS mice (317±67 mm^3^) and U87-0 mice (861±153 mm^3^) treated with 55.5 MBq ^188^Re for 4 weeks. Higher dose of ^188^Re did not increase therapeutic effect. Unlike thyroid cells, U87-hNIS cells are not polarized and therefore should express hNIS over all regions of their plasma membrane. In vivo, U87-hNIS tumors have a three-dimensional structure that places tumor cells in close proximity to each other. This geometry may allow rapid re-uptake of any isotope that leaks from one cell by surrounding cells and serve as a mechanism for isotope trapping by the tumor, which is in part responsible for the observed therapeutic effect of hNIS and ^188^Re in xenograft models. Therefore, cell arrangement can influence cytotoxicity. Studies [27] with hNIS cDNA transfected human glioma cells also showed increased cytotoxicity of ^131^I if cells were grouped in a three-dimensional spheroid culture compared to a monolayer culture. This was believed to be due to bystander toxicity, which is maximized in a three-dimensional model. As a corollary, to maximize the therapeutic effect of hNIS, high level transduction and expression are required. Thus, aiming for high level expression of hNIS makes sense not only for maximal radioisotope uptake but also to ensure adequate retention if the isotope is to have its desired effect.

In our study, little ^188^Re is retained in the thyroid gland, as ^188^Re cannot be organified by this organ. Studies have demonstrated a similar biodistribution pattern for^ 131^I and ^188^Re in mice, with the exception of the thyroid gland, in which only ^131^I is retained by organification. In fact, the absence of organification of ^188^Re by the thyroid gland may also be considered an advantage for therapy of nonthyroidal hNIS-bearing tissues [18], in that the thyroid will not serve as a sink for radiopharmaceuticals and will sustain less radiation damage, and more ^188^Re can be uptaken by U87-hNIS cells due to ^188^Re recirculation.

Considering that in our current study a stably hNIS transfected cell line was used with maximum hNIS expression levels, which is not directly applicable for clinical use in humans, the efficacy of ^188^Re needs to be evaluated further in future studies after systemic in vivo hNIS gene transfer with the typical limited transduction efficiency and a more heterogeneous hNIS expression pattern. Provided that these studies confirm our findings, ^188^Re may serve as an attractive alternative to ^131^I, particular in tumors with short iodide retention time.

## Conclusions

Our study is the first in vivo application of ^188^Re as an alternative radionuclide for the treatment of human glioma after lentivirus transfected sodium iodide symporter gene expression. Transfecting the hNIS gene by a lentiviral vector coupled with ^188^Re administration appears to be a novel and promising strategy for tumor imaging and therapy. Moreover, our study, based on the function of hNIS as a theranostic gene allowing the noninvasive imaging of hNIS expression by ^188^Re scintigraphy, provides detailed characterization of in vivo vector biodistribution and level, localization, and duration of transgene expression. These are essential prerequisites for precise planning and monitoring of clinical gene therapies that aim to individualize the hNIS gene therapy concept.
